# Unraveling functional decline: the relationship between muscle strength and ultrasound evaluation of biceps brachii thickness

**DOI:** 10.1007/s11357-025-01801-8

**Published:** 2025-07-18

**Authors:** Maria Chiara Brunese, Grazia Daniela Femminella, Leonardo Bencivenga, Francesco Tafuri, Corrado Caiazzo, Giuseppe Rengo, Germano Guerra, Klara Komici

**Affiliations:** 1 Department of Medicine and Health Sciences, University of Molise, Via F de Sanctis, 86100 Campobasso, Italy; 2 Department of Translational Medical Sciences, University of Naples “Federico II”, Naples, Italy; 3 Heracle Lab Research in Educational Neuroscience, Niccolò Cusano University, 00166 Rome, Italy; 4 Istituti Clinici Scientifici Maugeri IRCCS-Scientific Institute of Telese Terme, Telese Terme, BN Italy

**Keywords:** Muscle strength, Muscle ultrasound, Handgrip strength, Elderly, Muscle thickness

## Abstract

Skeletal muscle characteristics play a crucial role in understanding physical capacity, overall health, and outcomes. Muscle strength and muscle mass are key indicators of muscular function and sarcopenia. While previous research has explored biceps brachii muscle characteristics in isolation, there is a notable gap in comprehensive studies examining the direct correlation between biceps brachii muscle thickness (BMT), as measured by ultrasound, and muscle strength. In community-dwelling elderly individuals following physical capacity evaluations, anthropometric measurements, HGS, bioimpedance analysis, and muscle ultrasound for the measurement of biceps brachii muscle thickness were performed. The study population consisted of 156 participants; ICC for ultrasound measurements resulted in 0.96 and 95% CI 0.95–0.98, indicating excellent reliability. The Kruskal–Wallis analysis revealed that muscle thickness differed between age tertiles in both male (*p* = 0.0005) and female populations (*p* = 0.001). Regression analysis adjusted for age, sex, BMI, muscle mass in percentage, and comorbidities revealed a significant and independent positive correlation between muscle strength and muscle thickness: *R*^2^ = 0.70, *β* = 0.9, 95% CI: 0.7–1.2, and *p* < 0.001 in male population and *R*^2^ = 0.17, *β* = 0.2, 95% CI: 0.1–0.4, and *p* = 0.005 in female population. Muscle thickness of biceps brachii measured by ultrasound and handgrip strength is characterized by a significant and positive relationship in older adults. In both female and male populations, the reduction of muscle thickness is parallel to increased age. Implementation of noninvasive ultrasound evaluation of muscle thickness could be helpful in the early detection of physical decline associated with the aging process.

**Graphical Abstract:**

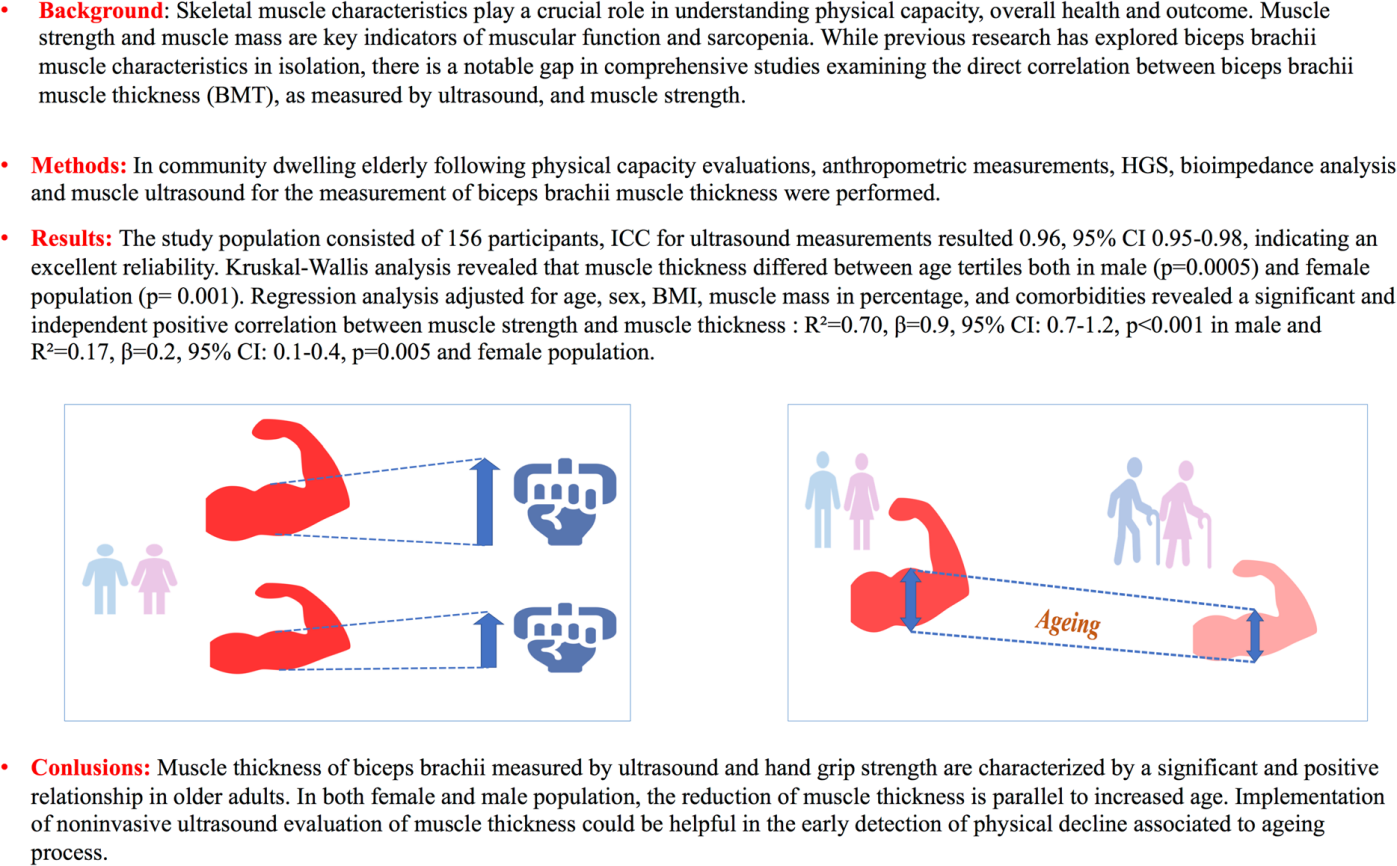

**Supplementary Information:**

The online version contains supplementary material available at 10.1007/s11357-025-01801-8.

## Introduction

Skeletal muscle characteristics play a crucial role in understanding physical capacity, overall health, and outcome [1, 2]. Muscle strength and muscle mass are key indicators of muscular function and sarcopenia [3]. Hand grip strength (HGS), measured through standardized dynamometric techniques, serves as a fundamental indicator of overall muscular strength and functional capacity [4]. This measurement has been widely recognized in clinical and research settings as a reliable proxy for assessing muscle performance and potentially predicting various health outcomes [5, 6]. Indeed, the association between physical activity and survival is lower in individuals with lower HGS [5]. Furthermore, HGS is a useful predictor of osteoporosis and falls [7]. HGS has been reported as an indicator of nutritional status and metabolic syndrome [8].

Ultrasound imaging has emerged as a non-invasive and reliable method for evaluating muscle morphology, providing precise evaluations with minimal discomfort to participants. During the last decade, ultrasound has gained attention for measuring muscle mass due to its real-time characteristics, availability, non-invasiveness, safety, and low cost. Some studies have suggested a significant role in the early detection of physical decline and frailty of muscle thickness measured by ultrasound [9, 10]. However, recent meta-analysis studies indicate limitations in population selection, muscles measured, and reference standards [11, 12]. As for the population selection, it should be mentioned that in a variety of studies measurements are performed in a rehabilitation setting, surgery, and patients with sepsis [13–15]. Additionally, despite the individual significance of biceps brachii muscle thickness and HGS, the potential relationship between these two parameters remains incompletely understood. While previous research has explored biceps brachii muscle characteristics in isolation [16], there is a notable gap in comprehensive studies examining the direct correlation between biceps brachii muscle thickness (BMT), as measured by ultrasound, and muscle strength. Therefore, in this study, we aimed to explore the relationship between BMT and muscle strength evaluated by HGS in a community-dwelling elderly population.

## Methods and materials

### Study population

The participants have been recruited among community-dwelling individuals following medical checkup at the Exercise and Sports Medicine Unit of “Antonio Cardarelli Hospital,” Department of Medicine and Health Sciences, University of Molise, Campobasso, Italy, from March 2024 till July 2024. For the purposes of this study, participants were considered eligible for enrollment if age was ≥ 60 years and could provide a written informed consent. Exclusion criteria were habitual vigorous physical activity training, wheel-chaired users, and inability to give written consent. The perceived exertion during vigorous physical activity on a scale of 0–10 points is 7–8, and examples of vigorous physical activity are running, tennis, basketball, football, swimming, and cycling ≥ 10 mph [17, 18]. The participants who reported at least 7 points on the perceived exertion scale and above-mentioned training disciplines were excluded. Assessments were performed on the same day of medical checkup. All procedures were approved by the Institutional Review Board of the Department of Medicine and Health Sciences, University of Molise, and conducted in accordance with the Declaration of Helsinki for studies on humans. All participants provided informed written consent for anonymous data collection prior to the study.

### Covariates

The presence of comorbidities including arterial hypertension, dyslipidemia, type 2 diabetes mellitus, ischemic heart disease, and chronic obstructive pulmonary disease (COPD) was assessed through patient anamnesis. These medical conditions were recorded as dichotomous variables (presence or absence) based on patient-reported physician diagnosis or current medication use. Assessment of these covariates was performed to account for potential confounding effects on muscle strength and muscle thickness measurements.

### Body composition analysis

A Wunder SA. Bl. srl A 200 stadiometer was used to measure height in meters, in a standing upright position. Weight, muscle mass, muscle mass percentage, fat mass, fat mass percentage, total body water in percentage, and bone mass were estimated by the electrical impedance body composition analyzer TANITA BC-420MA (Tanita Corporation, Tokyo, Japan), as indicated by the manufacturer. Body mass index (BMI) was also calculated.

### Measurement of muscle strength

Hand grip strength was evaluated with the JAMAR hydraulic dynamometer (Asimow Engineering, Los Angeles, CA, USA). Measurements were performed as described elsewhere [19]. Initially, participants sat on a chair with the shoulder in a neutral position, the elbow near the trunk and flexed at 90°, and the wrist with thumbs up. After a few minutes, they performed a maximal effort three times on both the right and left sides. Participants were asked to squeeze maximally for about 5 s, and the higher measurement was recorded in kilograms (kg). Reduced muscle strength was defined as HGS < 27 kg in males and < 16 kg in females, with values at or above these thresholds indicating preserved muscle strength [3].

### Biceps brachii muscle measurements

Ultrasound imaging was performed to obtain the assessment of biceps brachii muscle thickness (BMT). Measurements were conducted with participants in a standing position after 10 min of rest, on the dominant upper arm 60% distal between the lateral epicondyle of the humerus and the acromion process of the scapula. A linear array probe of an ultrasound diagnostic apparatus, Alpinion, was positioned perpendicular to the upper arm, and ultrasound gel was applied both on the probe and skin. After the image was registered, muscle thickness was measured as the distance from the adipose tissue–muscle interface to the muscle–bone interface three times, and the average was registered. For the purposes of our study, two physicians (M.C.B.) and (K.K.) performed measurements of BMT separately in a sample of 59 participants on the same day with the same methods. Measurements of BMT for the rest of the participants were assessed by K.K.

### Statistical analysis

The Shapiro–Wilk test was performed to explore the normality of data distribution. Data are expressed as median, interquartile range (IQR), number, and percentage. The interclass correlation coefficient (ICC) estimates and 95% confidence Interval were calculated by a two-way mixed-effects model and interpreted according to the following criteria: ICC > 0.90 excellent reliability, 0.75–0.90 good reliability, 0.50 to 0.75 moderate reliability, and poor reliability by ICC under 0.50 [20]. Sample size calculation for inter-rater reliability assessment was based on an expected ICC of 0.80 [21, 22], precision of 0.05, 95% confidence level, and 2 raters. This produced a required sample of 49 participants. Accounting for an anticipated 10% dropout rate, we planned to recruit at least 55 participants for the reliability substudy. Descriptive comparisons between groups were conducted according to sex and reduction of muscle strength. Continuous and categorical variables are compared with the Mann–Whitney *U*-test and chi-squared test with Fisher’s correction. Spearman’s correlation coefficient was used to test the correlations between age, HGS, and biceps brachii muscle thickness. Age was categorized in tertiles, and tertile division was chosen to capture the heterogeneity in muscle changes across age span. Previous research has reported an important increase in the prevalence of sarcopenia from 4% of men and 3% of women aged 70–75 to 16% of men and 13% of women aged 85 and older [23]. The Kruskal–Wallis analysis was performed to compare BMT values across age tertiles. Linear regression analysis was performed to evaluate the relationship between BMT and HGS, adjusted for confounders. Model assumptions for regression analysis were validated using the Shapiro–Wilk test for normality of residuals and the Ramsey RESET test for model specification [24, 25]. Goodness-of-fit was assessed using *R*^2^, with variable contributions expressed as percentages of the total explained variance. Multicollinearity among predictor variables was evaluated using variance inflation factors (VIFs). Values smaller than 4 have been suggested as the maximum acceptable VIF thresholds [25]. The statistical significance was *p* ≤ 0.05, and data were analyzed by STATA SE 16.1 (StataCorp LLC, College Station, TX, USA).

## Results

### General characteristics of population

A total population of 575 consecutive participants were screened, and 397 patients were excluded because of age < 60 years. Among 178 participants who fulfilled the age criteria, 22 participants were excluded because of the following reasons: participation in vigorous physical activity: 8; wheelchaired individuals: 4; incomplete data: 7; and did not agree to participate: 3 (Supplementary Materials, Fig s1). The final study population consisted of 156 participants, 49 (31.4%) males, 107 (68.5%) females, median age of 68.2 years, and IQR: 62.7–74.5. Inter-rater reliability was assessed in 59 participants (38% of total sample). ICC for ultrasound measurements resulted in 0.96, 95% CI: 0.95–0.98, *F* statistics: 37.9, and *p* < 0.001 indicating excellent reliability. The overall sample presented a median HGS of 19.3 kg, IQR: 15–20.6, and median BMT 20 mm, and IQR: 16–27. Female population presented higher BMI, lower muscle mass, lower fat mass, lower bone mass, lower HGS, and lower BMT (*p* < 0.05) compared to the male population. Characteristics of the population stratified by sex are reported in Table 1.

**Table 1:** Characteristics of population stratified by sex

	All populations *n* = 156	Male *n* = 49	Female *n* = 107	*p*-value
Age year, median, IQR	68.2 (62.7–74.5)	67.9 (62.1–75.3)	68.3 (63.2–73.6)	0.92
Hypertension, *n* (%)	67 (42.9)	15 (30.6)	48.6 (%)	0.03
Type 2 DM, *n* (%)	21 (13.5)	5 (10.2)	16 (14.9)	0.4
Dyslipidemia, *n* (%)	76 (48.7)	20 (40.8)	56 (52.3)	0.2
Smoking, *n* (%)	17 (10.1)	3 (6.1)	14 (13.1)	0.2
Ischemic heart disease, *n* (%)	9 (5.7)	4 (8.2)	5 (4.7)	0.5
COPD, *n* (%)	6 (3.8)	1 (2.1)	5 (4.7)	0.7
BMI kg/m2, *n* (%)	28.1 (25.4–31.6)	27.6 (25–28.8)	29.1 (25.5–32.4)	0.01
Muscle mass, kg, median, IQR	43.1 (39–51.1)	55 (48.9–60)	40.7 (38.1–44.3)	≤ 0.0001
Muscle mass, %, median, IQR	63.5 (57.6–69.4)	70.9 (68.6–74.6)	59.3 (54.8–64.2)	≤ 0.0001
Fat mass, kg, median IQR	22.2 (17.7–29.3)	19.9 (15.1–24.6)	25.4 (19–31.9)	0.0002
Fat mass, %, median IQR	32.9 (26.4–39.1)	24.6 (20.9–27.5)	36.9 (32.4–42.2)	≤ 0.0001
TBW%, mean SD	45.8 (42.3–50.5)	51.6 (49.6–54.2)	43.7 (40.8–46.1)	≤ 0.0001
Bone mass, mean SD	2.3 (2.1–2.4)	2.9 (2.6–3.1)	2.2 (2.1–2.4)	≤ 0.0001
HGS kg, mean, SD	19.3 (15–20.6)	32 (26–37)	17 (15–20.6)	≤ 0.0001
Biceps brachii muscle thickness mm, mean SD	20 (16–27)	32 (27–36.8)	18 (14–22)	≤ 0.0001

*IQR* interquartile range, type 2* DM *type 2 diabetes mellitus,* COPD *chronic obstructive pulmonary disease, *SD* standard deviation, *BMI* body mass index, *TBW* total body water, *HGS* handgrip strength

In univariate analysis, both male and female participants with reduced muscle strength resulted older than their counterparts with preserved muscle strength: 74.6 years, and IQR: 71.6–82.2 vs 63.2 years, IQR:61.8–74.7 (*p* = 0.01); 72 years, IQR: 68–76.6 vs 66.4 years, and IQR: 62.4–71.7 (*p* ≤ 0.0001). Biceps brachii muscle thickness was lower among male and female participants with reduced muscle strength as evaluated by HGS (*p* ≤ 0.0001 and *p* = 0.05). Table 2 summarizes the characteristics of the population stratified by HGS values in male and female participants.

**Table 2:** Characteristics of population stratified by reduction of muscle strength

	Reduced muscle strength Males (*n* = 13)	Preserved muscle strength Males (*n* = 36)	*p*-value	Reduced muscle strength Females (*n* = 35)	Reduced muscle strength Females (*n* = 72)	*p*-value
Age year, median, IQR	74.6 (71.6–82.2)	63.2 (61.8–74.7)	0.01	72 (68–76.6)	66.4 (62.4–71.7)	≤ 0.0001
Hypertension, *n* (%)	5 (38.5)	10 (27.8)	0.5	20 (57.1)	32 (44.4)	0.3
Type 2 DM, *n* (%)	1 (7.7)	4 (11.1)	0.7	3 (8.6)	13 (18.1)	0.2
Dyslipidemia, *n* (%)	4 (30.7)	16 (44.4)	0.5	19 (54.3)	37 (51.4)	0.8
Smoking, *n* (%)	0 (0)	3 (8.3)	0.6	4 (11.4)	10 (13.9)	0.7
Ischemic heart disease, *n* (%)	2 (15.4)	2 (5.6)	0.3	2 (5.7)	3 (4.2)	0.6
COPD, *n* (%)	0 (0)	1 (2.8)	0.7	1 (2.8)	4 (5.6)	0.5
BMI, kg/m^2^ median, IQR	28.3 (25.5–32.4)	27.5 (24.9–28.2)	0.3	29.2 (27.1–32.8)	28.8 (25.5–32.3)	0.4
Muscle mass, kg/m^2^ median, IQR	52.1 (47.1–58.6)	55.8 (50.9–60)	0.4	39.3 (37.6–43.9)	40.8 (38.8–44.4)	0.1
Muscle mass, %, median, IQR	70.1 (64.8–72.3)	71.6 (69.1–74.9)	0.3	59.7 (56.4–64.2)	60.6 (54.8–64.1)	0.06
Fat mass, median, IQR	18.9 (13.6–26.3)	26.9 ± 3.2	0.9	25.7 (18.5–30.5)	25.2 (19.5–32.1)	0.6
Fat mass, %, median, IQR	25.6 (19.3–29.3)	24.5 (21.0–27.2)	0.8	36.9 (32.3–40.6)	36.1 (32.5–42.2)	0.9
TBW% median, IQR	51.7 (48.8–54.0)	51.5 (50–54.2)	0.8	43.8 (40.9–45.5)	43.6 (40.5–46.6)	0.8
Bone mass, median, IQR	2.8 (2.5–3)	3 (2.7–3.1)	0.3	2.1 (2–2.3)	2.2 (2–2.4)	0.1
HGS kg, median, IQR	19 (16–21.6)	34.8 (31.1–39.5)	≤ 0.0001	13.7 (12–15)	19.3 (17–22)	≤ 0.0001
Biceps brachii muscle thickness, mm, mean SD	24 (19–28)	34.2 (30–38.6)	≤ 0.0001	16 (14–19)	19 (15.5–22)	0.05

*SD* standard deviation, *BMI* body mass index, *TBW* total body water, *HGS* handgrip strength, *IQR* interquartile range

### Muscle thickness and age

**Fig. 1:**
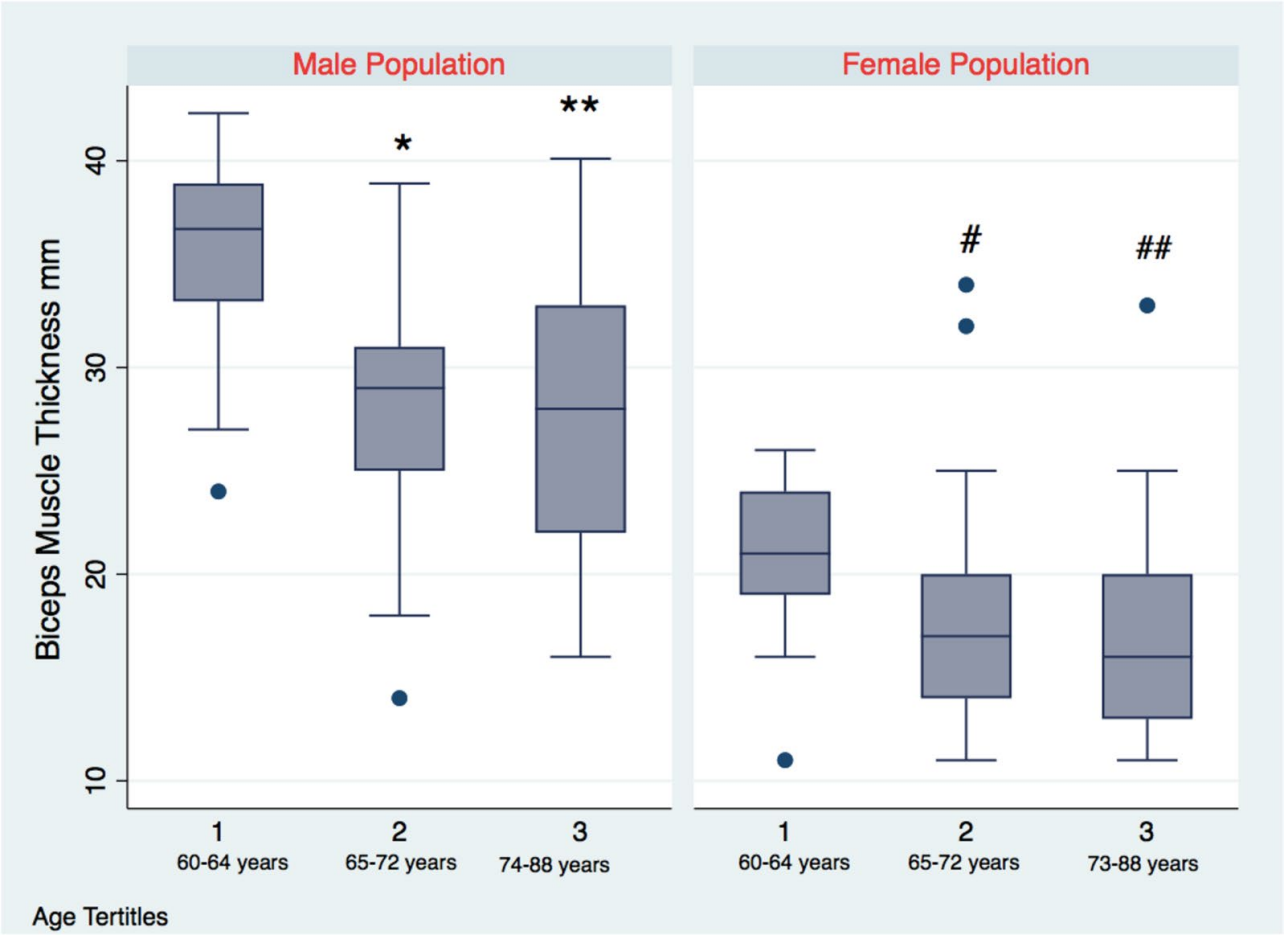
Biceps brachii muscle thickness across age tertiles age ranges are provided for each tertile. *p = 0.005 1 vs 2 tertile; **p = 0.001 1 vs 3 tertile; #*p* = 0.0005 1 vs 2 tertile; ##*p* = 0.0003 1 vs 3 tertile

Spearman’s correlation coefficient showed that BMT was negatively correlated with age (males: *ρ* =  − 0.47 and *p* = 0.0005; females: *ρ* =  − 0.42 and *p* = 0.001) For the male population, age tertiles were as follows: tertile 1 (60–64 years), tertile 2 (65–72 years), and tertile 3 (74–88 years). For the female population, tertiles were as follows: tertile 1 (60–64 years), tertile 2 (65–72 years), and tertile 3 (73–88 years). The Kruskal–Wallis analysis revealed that muscle thickness differed between age tertiles both in male (*p* = 0.001) and female populations (*p* = 0.0002), and the pairwise analysis showed a significant difference between the first and the second tertile and the first and the third in male and female population: *p* = 0.0005 and 0.0003 for the female population and *p* = 0.005 and *p* = 0.001 for the male population, respectively (Fig. 1). Regression analysis in the male population, adjusted for BMI, muscle mass percentage, and comorbidities such as arterial hypertension, diabetes mellitus type 2, dyslipidemia, chronic obstructive pulmonary disease, and ischemic heart disease, revealed a significant and independent positive correlation between age and biceps brachii thickness: *R*^2^ = 0.22, *β* =  − 0.4, and *p* < 0.001. The same analysis in female population resulted significant and independent: *R*^2^ = 0.19, *β* =  − 0.2, and *p* = 0.001 (Supplementary Materials Tab s1). The contribution of age to the total *R*^2^ for male and female population was, respectively, 26.3% and 11.6%. In both populations, the Shapiro–Wilk test and Ramsey RESET test resulted not significant: *p* = 0.3 and *p* = 0.4 for the male population and *p* = 0.1 and *p* = 0.8 for the female population. Multicollinearity was also negative for the male and female populations: VIF = 1.16 and VIF = 1.09.

### Muscle thickness and muscle strength

**Fig. 2:**
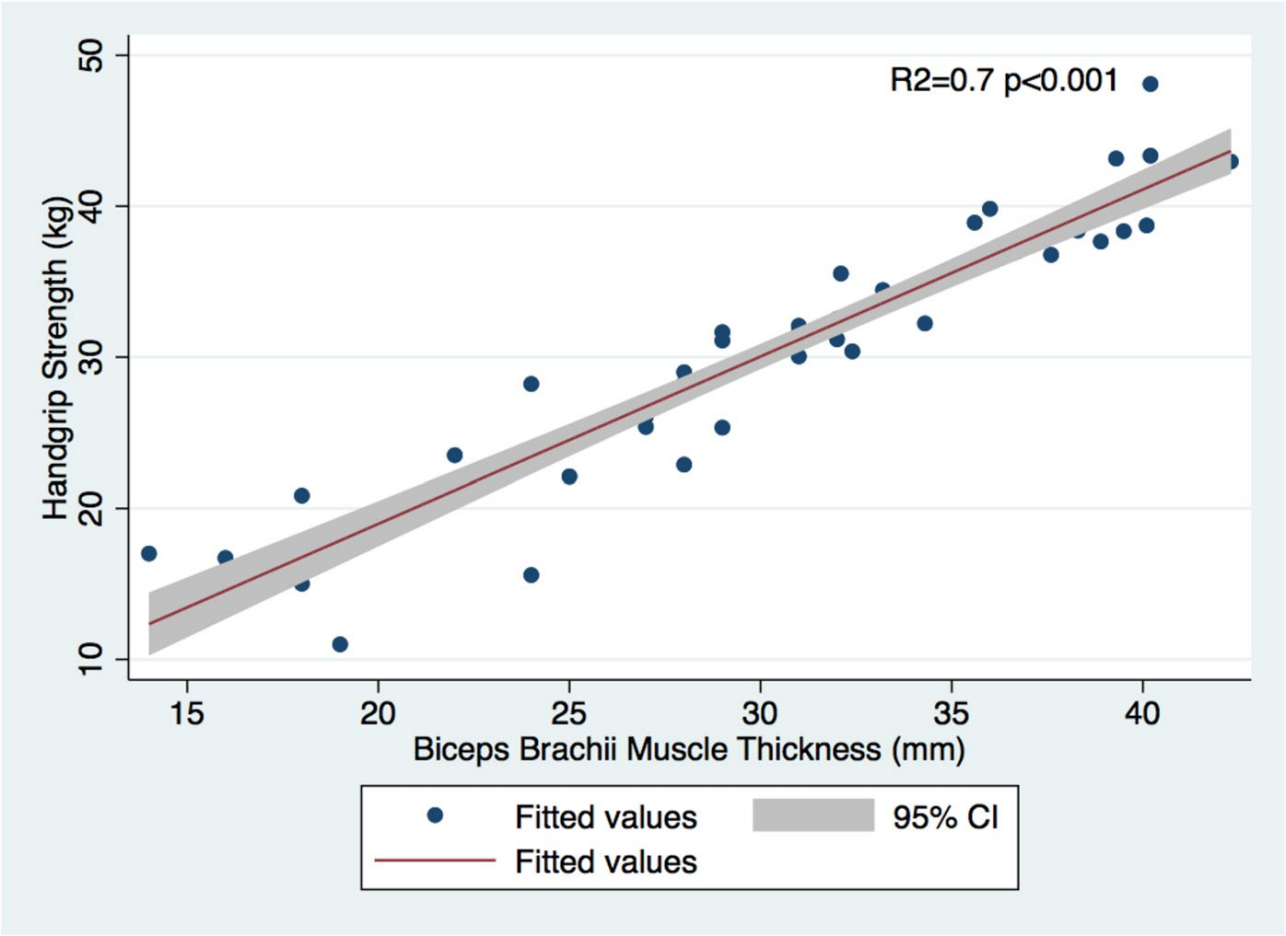
Relationship between HGS and biceps brachii muscle thickness in male population

**Fig. 3:**
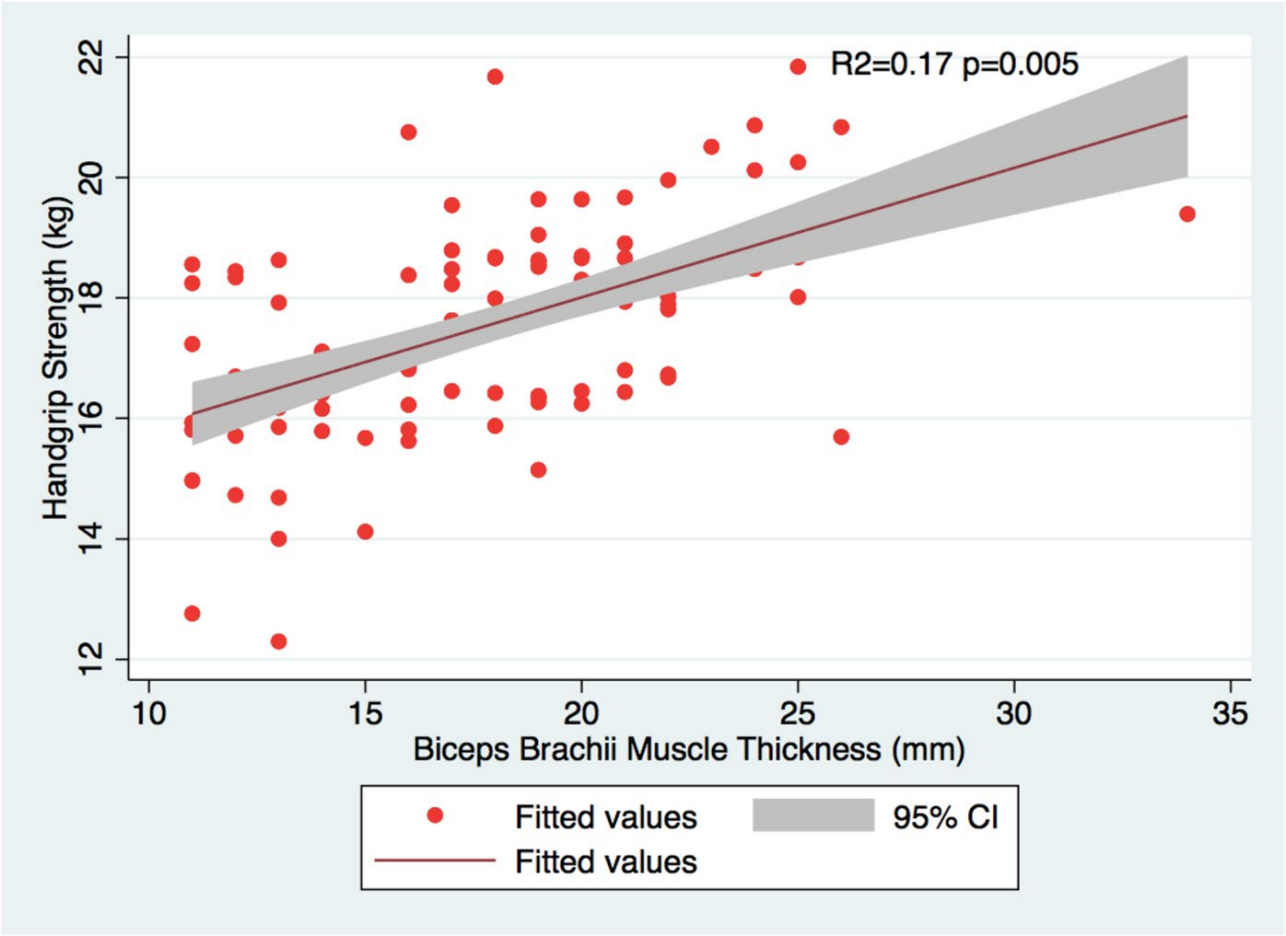
Relationship between HGS and biceps brachii muscle thickness in female population

The Spearman’s correlation coefficient showed that BMT was positively correlated with HGS (males: *ρ* = 0.86 and *p* < 0.0001; females: *ρ* = 0.25 and *p* = 0.007). Regression analysis in the male population, adjusted for age, BMI, muscle mass percentage, and comorbidities such as arterial hypertension, diabetes mellitus type 2, dyslipidemia, COPD, and ischemic heart disease, revealed a significant and independent positive correlation between muscle strength and biceps brachii thickness: *R*^2^ = 0.70, *β* = 0.9, 95% CI: 0.7–1.2, and *p* < 0.001. Biceps thickness contributed 36% to the total *R*^2^, with a VIF of 1.7 indicating no significant multicollinearity. In the female population, the correlation was also statistically significant: *R*^2^ = 0.17, *β* = 0.2, 95% CI: 0.1–0.4, and *p* = 0.005, with biceps thickness contributing 7% to the total *R*^2^ and a VIF of 1.1 confirming low multicollinearity (Figs. 2 and 3).

In both male and female populations, the Shapiro–Wilk test confirmed that the residuals followed a normal distribution, satisfying the normality assumption required for reliable statistical inference: *p* = 0.9 and *p* = 0.6. Subsequently, the Ramsey RESET test did not indicate significant results: male population (*p* = 0.4) and female population (*p* = 0.7). These diagnostic results support the adequacy of the linear regression model and suggest that the assumptions underlying the analysis are appropriately met. Histogram residuals and Q-Q plots are presented in Supplementary Materials Figs. 2–5.

## Discussion

In the present study, we found a significant and independent relationship between biceps brachii muscle thickness measured by ultrasound and muscle strength in a population of community-dwelling older adults. In both female and male populations, the reduction of muscle thickness is parallel to increased age.

Muscle ultrasound has emerged as a promising method in the identification of muscle quantity and quality [26]. The application of muscle ultrasound has been explored in various and heterogeneous acute and chronic clinical contexts such as stroke [22, 27], COPD [28], coronary artery disease patients [29], and pre- and post-operative osteoarthritis [30]. Few studies have applied this method in community-dwelling adults [21]. Furthermore, the methods for muscle ultrasound measurements are heterogeneous, and there is currently a lack of a clear and standardized protocol.

In a recent systematic review and meta-analysis study, Fu et al. [12] underlined that only 41% of the studies focused on muscle ultrasound evaluation and sarcopenia reported data regarding intra- and inter-rater reliability to determine agreement between the scans or raters. In our study, measurement of muscle thickness by ultrasound is characterized by excellent reliability, as demonstrated by ICC of 0.96. The significant and independent correlation between muscle thickness and muscle strength is the most important result of our study. This relationship remained significant even after adjustment for potential confounding variables, such as age and comorbidities, indicating that muscle thickness measured by ultrasound may serve as an independent predictor of muscle strength. Previous studies have reported that muscle thickness of forearm ulnar and dorsal interosseous resulted as factors contributing to the prediction of handgrip strength in young adults [31], and loss of adductor and quadriceps muscles is associated with zig-zag walking [31] suggesting a relationship with muscle strength. Furthermore, in adults with cerebral palsy and severe motor involvement, a reduced thickness of vastus intermedius and rectus femoris was found compared to patients with moderate motor involvement [32], suggesting that muscle thickness could be used as a surrogate of muscle strength.

Analysis across age tertiles demonstrated a progressive decline in muscle thickness with advancing age in both male and female populations. This age-related decline was evident when comparing the youngest tertile (60–64 years) to the middle tertile (65–72 years) and to the oldest tertile (over 72 years) in both sexes. Linear regression analysis revealed a significant and independent relationship between age and muscle thickness, indicating that biceps brachii thickness continues to decrease substantially with advancing age within the older adult population. The observed reduction in muscle thickness with age aligns with established patterns of age-related muscle mass decline. However, our findings extend this knowledge by demonstrating that significant muscle thickness reduction continues to occur suggesting ongoing muscle architectural changes throughout later life. The reduction in muscle thickness with age in both sexes can be attributed in part to age-related changes in muscle composition and architecture. Progressive muscle mass decline, which accelerates after 60 years of age [33], may contribute to the reduced muscle thickness observed in our study. Concurrent redistribution of adipose tissue and increased intramuscular fat infiltration [34, 35] may contribute to the modification of muscle fibers organization. Indeed, skeletal muscle ectopic fat infiltration increases with aging and is recognized to negatively correlate with muscle strength and muscle mass [36].

Biceps brachii muscle thickness differed significantly between female and male populations. Our observation aligns with other studies which reported sex-related differences regarding ultrasound characteristics of vastus lateralis and rectus femoris [37, 38]. The variations can be attributed to multiple factors, including muscle fiber composition, hormonal differences, and typical physical activity patterns. Males are characterized by larger fiber cross-sectional areas, more similar to type II fiber characteristics, whereas females have smaller fibers similar to type I characteristics [39]. Of interest, a sex-specific association between adiponectin and frailty has been suggested by a recent study [40], and myokines and adiponectin have been suggested as possible biomarkers for sarcopenia [41]. Testosterone activates muscle protein synthesis by triggering the Akt/mTOR pathway, inhibits muscle protein degradation, and promotes the recruitment of mesenchymal pluripotent stem cells into the myogenic lineage [42–45].

The design of our study is cross-sectional and we are not able to establish the causal-effect relationship between muscle strength and muscle thickness. It has been reported that high-intensity upper and lower resistance training resulted in a significant increase of triceps muscles after 6 weeks while muscle strength improved after 12 weeks of exercise [46].

Another study reported that after 10 weeks of resistance training increased both muscle size and strength but the effects of detraining were more pronounced in muscle size [47]. Perceived neural adaptations, motor learning ability of the central nervous system, and the re-sensitization of hypertrophic signaling in myofibers have been purposed as underlying mechanisms [48]. However, further research is necessary to establish this bidirectional relationship, and longitudinal investigations are necessary to clarify the pathways linking muscle morphology and functional capacity.

Although in our study the relationship between muscle strength and muscle thickness was analyzed considering potential confounders such as cardiovascular risk factors and COPD, other age-related comorbidities and unexplored factors may influence this relationship. Low muscle strength is found in approximately 43% of patients with chronic kidney disease [49]; muscle ultrasound has been suggested as a valuable method for identifying sarcopenia in patients with CKD [50]. Anterior thigh muscle thickness resulted significantly associated with osteoporosis [51], and quadriceps muscle thickness was reduced in sarcopenic patients with rheumatoid arthritis [52]. Additionally, vitamin D deficiency [53] and drugs may influence the interaction between muscle strength and thickness [54]. Future studies should consider the implication of low-grade inflammation, oxidative stress, and insulin resistance in mediating the relationship between muscle architecture and function in older adults.

In the field of prevention medicine, understanding the relationship between muscle thickness and strength can help in the early detection of age-related physical decline and in monitoring training strategies. Furthermore, in the rehabilitation setting, the precise measurement techniques and correlations can guide more targeted muscle assessment and recovery protocols.

Our study is characterized by some limitations. First, this is a single-center study with a limited number of participants. Second, potential impacts of lifestyle factors are not fully captured, and further longitudinal studies are necessary to validate age-related muscle changes. Third, the cross-sectional design is another limitation of our study, as we cannot determine cause-effect associations between muscle strength and muscle thickness.

Finally, we did not compare muscle ultrasound with other imaging methods such as computed tomography (CT) or magnetic resonance imaging (MRI). However, previous studies have confirmed the validity of muscle ultrasound in quantifying muscle size through comparisons with these techniques [29, 55–57]. Several factors support the use of ultrasound: CT and MRI are characterized by limited accessibility, are time-consuming, and are more expensive compared to ultrasound [26]. Additionally, CT involves radiation exposure, which may raise ethical concerns [26]. For studies involving elderly populations and large-scale screening, muscle ultrasound offers significant advantages as it is widely used in clinical practice, is noninvasive, is safe for patients, and is familiar to clinicians.

## Conclusions

Muscle thickness of biceps brachii measured by ultrasound and hand grip strength are characterized by a significant and positive relationship in older adults. In both female and male populations, the reduction of muscle thickness is parallel to increased age. Implementation of noninvasive ultrasound evaluation of muscle thickness could be helpful in the early detection of physical decline associated with the ageing process. However, the validity of the muscle ultrasound against other reference imaging methods regarding muscle quantity and quality and the development of a standardized protocol remains imperative to the strength of future research.

## Supplementary Information

Below is the link to the electronic supplementary material.

Supplementary file1 (DOCX 13845 KB)

## Data Availability

The dataset analyzed during the current study are available from the corresponding author on reasonable request.
