# Supplementary material for: Unraveling functional decline: the relationship between muscle strength and ultrasound evaluation of biceps brachii thickness
Source: GeroScience. 2025 Jul 18;48(2):2479–90. doi: 10.1007/s11357-025-01801-8 (PMC12972363; doi:10.1007/s11357-025-01801-8)
Supplement: Supplementary file 1 — Supplementary file1 (DOCX 13845 KB) [file 11357_2025_1801_MOESM1_ESM.docx]

Supplementary Material. Figure 1. Flow diagram of the population screening.

|  | Male Population | |  | Female Population | | |
| --- | --- | --- | --- | --- | --- | --- |
| Variables | Coefficient | 95% CI | p-value | Coefficient | 95% CI | p-value |
| Age | -0.45 | -.67 -.20 | <0.001 | -0.24 | -.38 -0.10 | 0.001 |
| BMI | -0.46 | -1.0 0.11 | 0.1 | 0.20 | -.01 0.42 | 0.06 |
| Muscle Mass % | 0.02 | -0.16 0.21 | 0.8 | 0.15 | -0.01 0.33 | 0.07 |
| DM type 2 | 6.13 | -0.25 12.53 | 0.1 | -0.78 | -3.3 1.8 | 0.53 |
| Arterial Hypertension | .3817135 | -4.18 4.9 | 0.8 | -0.08 | -2.06 1.89 | 0.93 |
| Ischemic Heart Diseased | -2.63 | -10.8 - 3.9 | 0.3 | -0.96 | -8.07 0.18 | 0.67 |
| Dyslipidemia | -3.02 | -6.9 0.92 | 0.1 | -0.87 | -2.74 1.00 | 0.35 |
| COPD | 7.46 | -7.84 18.2 | 0.4 | -3.94 | -3.39 1.83 | 0.07 |

Supplementary Material Table 2. Regression analysis adjusted for confounders

95% CI: 95% Confidence Interval; DM: Diabetes Mellitus; COPD: Chronic Obstructive Pulmonary Disease; BBMT: Biceps Brachii Muscle Thickness


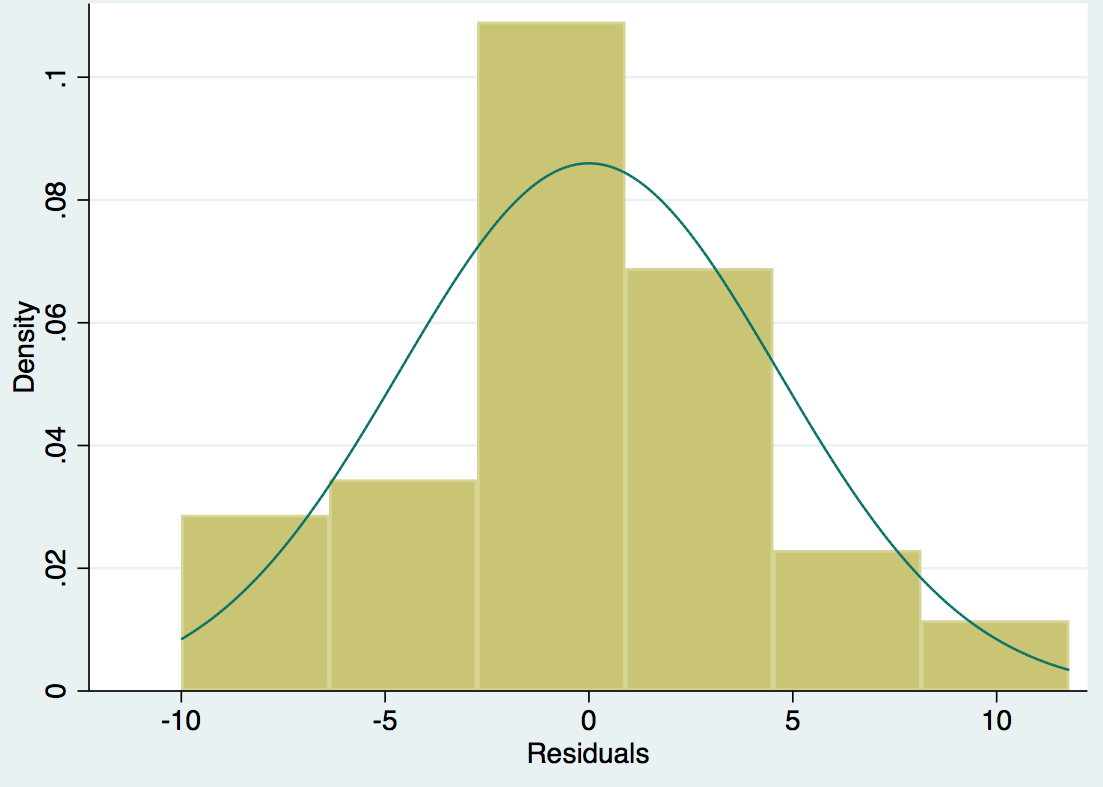


Figure 2. Histograms normality in Male population


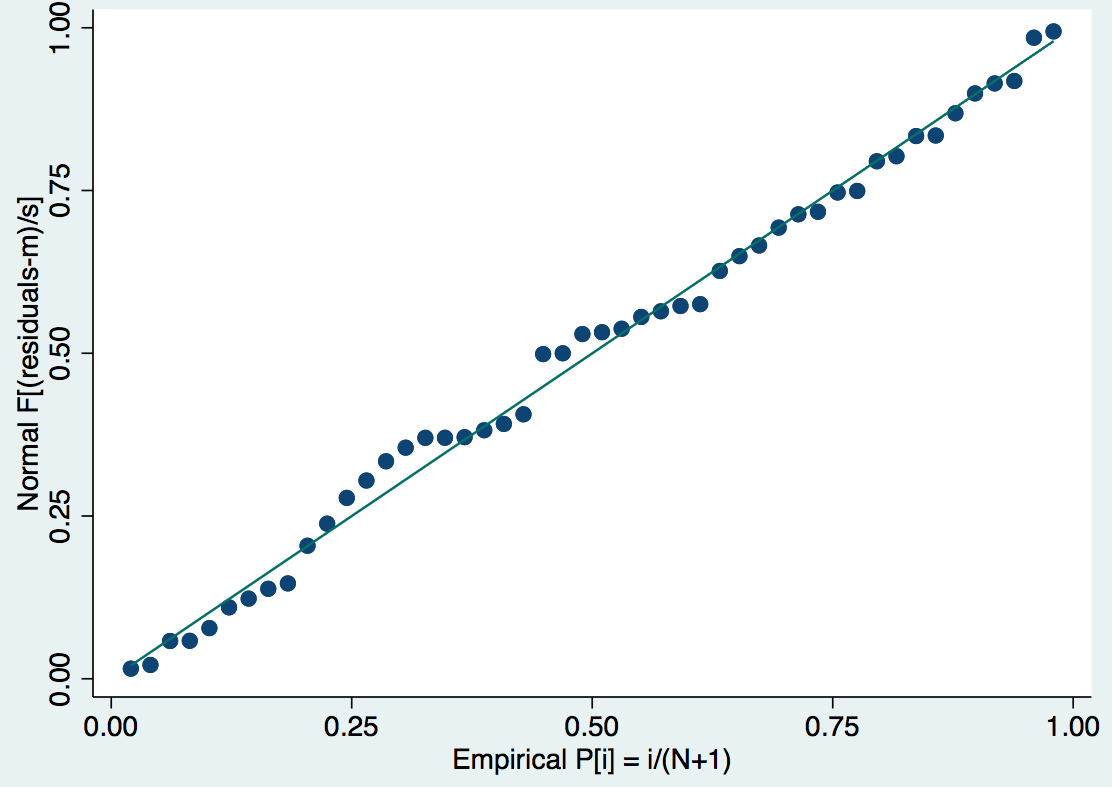


Figure 3. Q-Q plots in Male Population


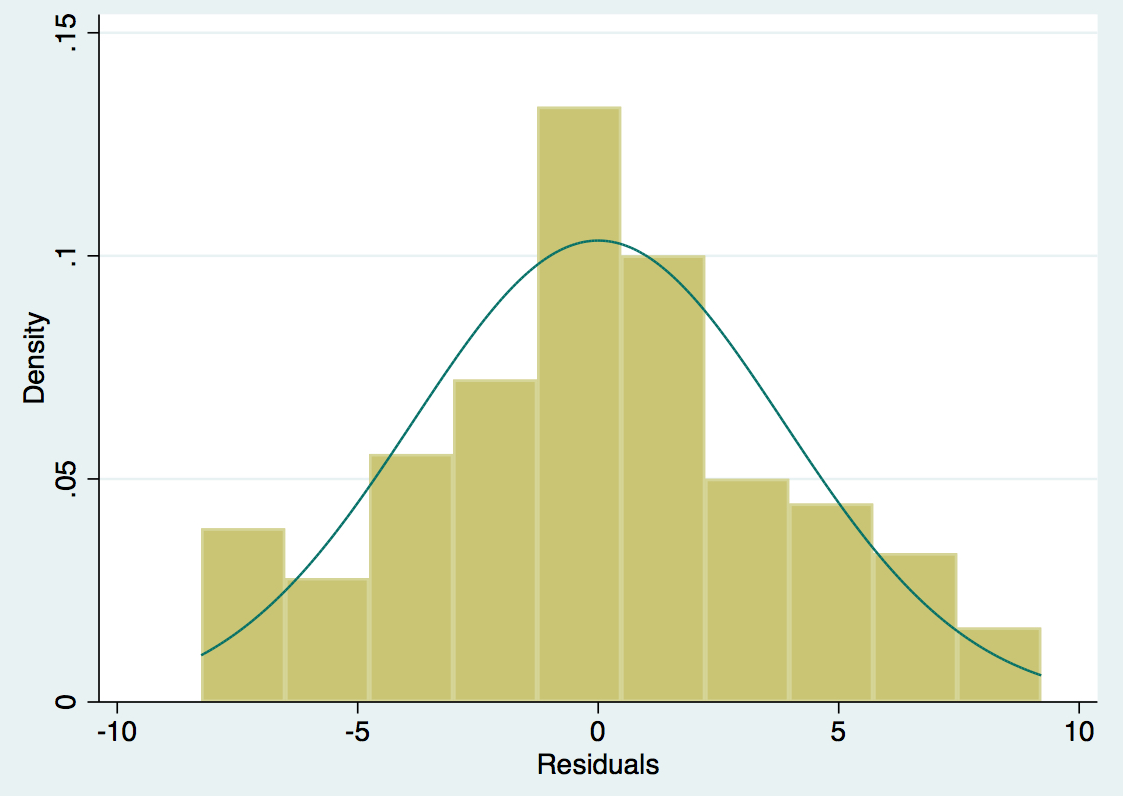


Figure 4. Histograms normality in Female Population


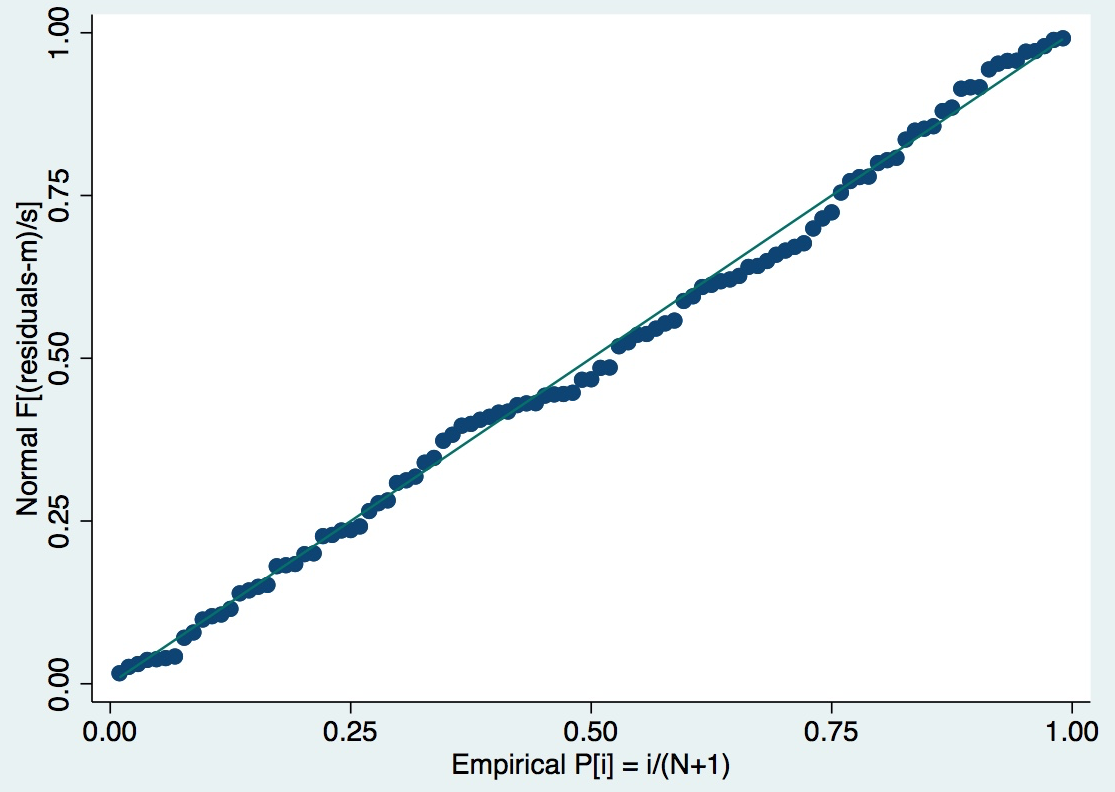


Figure 5. Q-Q plots in Female Population
